# Pancreatic Cancer Cell Exosome-Mediated Macrophage Reprogramming and the Role of MicroRNAs 155 and 125b2 Transfection using Nanoparticle Delivery Systems

**DOI:** 10.1038/srep30110

**Published:** 2016-07-22

**Authors:** Mei-Ju Su, Hibah Aldawsari, Mansoor Amiji

**Affiliations:** 1 Department of Pharmaceutical Sciences, School of Pharmacy, Northeastern University, Boston, MA 02115, USA; 2 Faculty of Pharmacy, King Abdulaziz University, Jeddah, Saudi Arabia

## Abstract

Exosomes are nano-sized endosome-derived small intraluminal vesicles, which are important facilitators of intercellular communication by transporting contents, such as protein, mRNA, and microRNAs, between neighboring cells, such as in the tumor microenvironment. The purpose of this study was to understand the mechanisms of exosomes-mediated cellular communication between human pancreatic cancer (Panc-1) cells and macrophages (J771.A1) using a Transwell co-culture system. Following characterization of exosome-mediated cellular communication and pro-tumoral baseline M2 macrophage polarization, the Panc-1 cells were transfected with microRNA-155 (miR-155) and microRNA-125b-2 (miR-125b2) expressing plasmid DNA using hyaluronic acid-poly(ethylene imine)/hyaluronic acid-poly(ethylene glycol) (HA-PEI/HA-PEG) self-assembling nanoparticle-based non-viral vectors. Our results show that upon successful transfection of Panc-1 cells, the exosome content was altered leading to differential communication and reprogramming of the J774.A1 cells to an M1 phenotype. Based on these results, genetic therapies targeted towards selective manipulation of tumor cell-derived exosome content may be very promising for cancer therapy.

By 2030, pancreatic cancer is predicted to become the second leading cause of cancer-related deaths in the United States and many other parts of the world^1^. Pancreatic ductal adenocarcinoma (PDAC) constitutes the majority (80–90%) of cases of pancreatic cancer^2^. PDAC is an aggressive malignancy of the exocrine pancreas with a 5-year survival rate of ranges from 8–25%^3^. Due to the majority of patients initially present with advanced and metastatic disease with less than 10% of patients are diagnosed with pancreatic cancer at early-stages, the new strategies to treat pancreatic cancer are sorely needed for the vast majority of patients with PDAC^4^,^5^. One hallmark of PDAC is the pronounced massive tumor stroma^6^. This non-neoplastic inflammatory stromal microenvironment can further promote the initiation and progression of PDAC and is mainly composed of extracellular matrix, activated carcinoma-associated fibroblasts, and various immune cells such as regulatory T cells and tumor-associated macrophages (TAMs), which may play an important role in a process called epithelial-mesenchymal-transition (EMT), involved in early tumorigenesis^7^,^8^.

Tissue-associated macrophages are derived from the myeloid progenitor cells and infiltrate various parts of the body including solid tumors^9^. Both inflammatory and resident macrophages perform a range of essential biological functions and are activated in response to environmental signals, including microbial products and cytokines^10^,^11^. Activated macrophages possess phenotypic plasticity that can be divided into a continuum of M1 and M2 functionally polarized states^12^. M1 macrophages, also known as classically activated macrophages, play various roles in both innate and adaptive immune system^9^. Classical activation to M1 macrophages occurs in response to bacterial moieties, such as lipopolysaccharide (LPS) and immune stimuli, such as interferon γ (IFNγ)^8^. In contract, M2 macrophages, also known as alternatively activated macrophages, are better adapted to scavenging debris and release growth factors that promote angiogenesis and fibrosis^11^,^13^,^14^. Even though they are still highly phagocytic, the main roles of M2 macrophages is helping repair sites of injury by engulfing cell debris, regulating tissue re-modeling, and repair or control normal cell turnover^9^. Alternative activation to M2 macrophages comes in different varieties depending on the eliciting signals, including IL-4, IL-13, IL-10, and glucocorticoid hormones^15^.

The myeloid cells that infiltrate into the tumor stroma usually differentiate into TAMs and predominantly express the M2 phenotype^16^. TAMs also produce a wide range of pro-angiogenic factors, including endothelial growth factors and extracellular matrix remodeling proteases and immunosuppressive factors^9^. The macrophage inflammatory protein-3 alpha (MIP-3α), which is expressed by pancreatic cancer cells and TAMs have been implicated as a regulator of tumor cell invasion in human pancreatic cancer^17^,^18^. Therefore, since tumor promoting TAMs are predominantly of the M2 phenotype in the case of solid tumors, including PDAC, reprogramming of them towards predominant anti-tumoral M1 phenotype may holds great promises in the effective treatment of cancers^19^,^20^.

Exosomes are endosome-derived small intraluminal vesicles that are characterized by the size range from 30 nm to 100 nm in diameter and the density range from 1.13 g/ml to 1.19 g/ml in a sucrose gradient with a flattened sphere limited by a bi-lipidic layer^21^,^22^. Although they were first found in maturing red blood cells, these vesicles can be generated by a large number of cell types including tumor cells, neurons and immune cells, and also detected in body fluids such as serum and plasma^23^. Typical exosomes express major histocompatibility complex MHC I and MHC II on their surface and contain certain specific marker tetraspanins, such as CD9, CD63, CD81, heat-shock proteins, lipids and miRNAs^24^,^25^. Increasing scientific evidence indicates that exosomes play an important role and act as mediators in cell-cell communication^26^,^27^,^28^. Previous studies have shown that tumor cells secrete excessive amounts of exosomes compared to normal cells^29^. By secreting exosomes, tumor cells can re-program cells in the tumor microenvironment with the aim to promote tumor initiation, invasion and metastasis^29^,^30^.

MicroRNAs are small non-coding RNAs with diverse functions, which can regulate gene expression at the post- translational level by binding to the 3′ untranslated-region (UTR) of their target mRNAs to repress translation or direct cleavage^31^. The biogenesis of miRNAs is tightly controlled, and dysregulation of miRNAs is linked to cancer^32^,^33^,^34^. MiR-155 is a multifunctional microRNA that possess crucial functions in hematopoiesis, inflammation, cancer and immunity^35^,^36^. MiR-155 is up-regulated in response to lipopolysaccharides (LPS) or interferon (IFN) signaling in both macrophages and monocytes of mouse or human origin, suggesting that this microRNA plays crucial roles in the innate immune response to both viral and bacterial infections^37^. Additionally, it has been proven that miR-155 has a critical role in macrophage polarization, as the knockdown resulted in the transitioning of macrophages to a M2/Th2 response^38^. MiR-125b is a ubiquitously expressed microRNA that is aberrantly expressed in a great variety of tumors^39^,^40^,^41^. It has been shown when miR-125b is overexpressed in macrophages, it enhances surface activation markers in response to interferon gamma (IFN-γ)^42^. MiR-125b is enriched in M1 phenotype macrophage and has been associated with improved antigen presentation, enhanced T-cell activation and tumor destruction^43^. The action of miR-125b is via inhibition of interferon regulatory factor-4, which is a negative regulator of pro-inflammatory macrophage activation^39^,^43^.

In this study, we hypothesized that transfection of PDAC cells with miR-encoding plasmid DNA will affect the exosomal content and subsequently change the macrophage polarization from M2 to M1. We have investigated the effect of miR-155 and miR-125b transfection using CD44 targeting hyaluronic acid (HA)-based self-assembling nanoparticles^44^. HA is a highly anionic biopolymer present in the extracellular matrix and synovial fluids, which have emerged as promising nanocarriers for targeting genes or drugs to tumor cells that over-express CD44 receptor^14^,^44^,^45^,^46^. With the modification of the sugar residues with variety of functional macrostructures on HA polymer is able to facilitate self-assembly and encapsulation of diverse gene payloads for targeted delivery^47^. Although HA is an ideal carrier polymer, its anionic nature can have difficulty in encapsulating negatively charged siRNA molecules^44^. Hydrophobic modification of HA backbone may be one of the solutions to overcome the limitation, with fatty amines and cationic polyamines that would not only help reduce the net negative charge density on the backbone polymer, but also facilitate gene payloads encapsulation via self-assembly^48^. HA was further chemically conjugated with poly(ethylene imine) (PEI) and with poly(ethylene glycol) (PEG), and the outcome of some studies indicated that the HA-nanocarrier system made by blending HA-PEI/HA-PEG blocks was ideal for delivery of nucleic acid constructs (e.g., siRNA) to solid tumors and showed the highest target gene knockdown as compared to the other conditions that had either HA-PEI alone with siRNA or with all the three HA components with siRNA^44^,^49^,^50^.

We have synthesized HA-PEI and HA-PEI conjugate for encapsulation and specific delivery of plasmid DNA expressing miR-155 and miR-125b to panc-1 cells for modulation of their exosomes cargo in order to achieve anti-tumoral effect on M1 macrophage via exosomes-mediated reprogramming.

## Results

### Establishment of Panc-1 and J774.A1 Macrophages in Transwell Co- culture System

**Figure 1:**
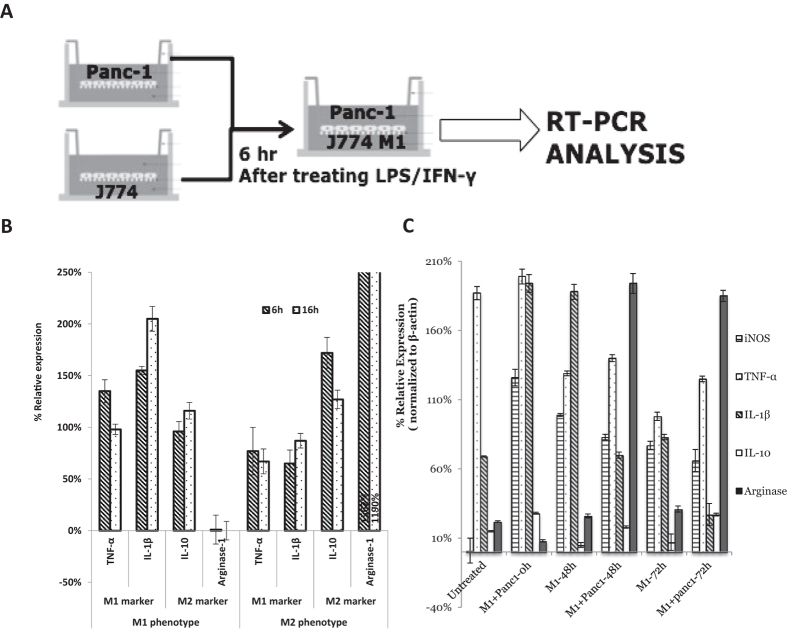
Macrophage Polarization Studies using an Indirect Transwell Co-Culture System. (**A**) Schematic illustration of indirect co-culture system. (**B**) RT-PCR quantitative analysis of M1 and M2 specific gene expression after 6 h/16 h LPS + IFN γ –stimulation and IL-4 stimulation (100 ng/ml). The expression level was normalized to untreated J774A.1 cells. β-actin was used as endogenous housekeeping gene. (**C**) RT-PCR quantitative analysis of M1 and M2 specific gene expression in co-culture system at 48 h and 72 h. The J774.A macrophages that did not receive any treatment were labeled as “untreated” in the graph. The J774.A macrophages that received LPS and IFN-γ were labeled as “M1 + Panc-1-0h”. Comparing with the untreated cells, the M1 + Panc-1-0h group showed the increase in expression of M1 markers and decrease in M2 marker compared to the control group, which indicated that the J774A.1 macrophages were successfully polarized to M1 state after LPS and IFN- γ treatment. Two groups of J774A.1 macrophages in M1 state were further co-cultured with Panc-1 cells for 48 h and 72 h. The polarity of the two J774A.1 macrophages groups changed to M2-polarized phenotype as indicated by an increase in expression of M2 markers and decrease in M1 markers compared to M1 groups.

Before creating the indirect co-culture system for evaluating J774A.1 macrophages polarity in tumor microenvironment, we firstly establish a system for macrophage polarization studies to evaluate baseline macrophage polarity. J774A.1 macrophages were stimulated with different stimuli, LPS and IFN-γ or IL-4 cytokine, and the expression level of specific M1 and M2 markers were determined by RT-PCR. Stimulation of J774A.1 macrophages with LPS and IFN-γ for 6 h or 16 h induced M1 macrophages with high level of TNF-α and IL-1β while the expression of Arg1 and IL-10 were downregulated. A simultaneous increase in expression of M1 markers and decrease in M2 markers confirmed polarization of macrophages to the M1 phenotype. In contrast, J774A.1 stimulated with IL-4 cytokine for 6 or 16 h upregulated the expression of Arg and IL10 with low expression of TNF-α and IL-1β comparing to M1 phenotype (Fig. 1b). A simultaneous increase in expression of M2 markers and decrease in M1 markers confirmed polarization of macrophages to the M2 phenotype after stimulating with IL-4 cytokine. Since stimulating J774A.1 macrophages with different stimuli for 6 hours has shown the effect of polarity changes, 6 h polarization had been used for all the future studies when J774A.1 macrophages needed to be polarized.

In a Transwell co-culture system, J774A.1 macrophages were seeded at the bottom of the Transwell system and stimulate the cells with LPS and IFN-γ with the aim of promoting the M1 phenotype of macrophages. Pancreatic cancer cells (Panc-1) were seeded into the upper chamber of the Transwell insert. The culture inserts with Panc-1 cells were placed into the culture dishes containing J774A.1 macrophages after macrophages were polarized to M1 phenotype and incubated for 48 h/72 h (Fig. 1a). After co-culture with Panc-1cell, the phenotype of J774A.1 cells had been analyzed by RT-PCR with specific M1 markers (iNOS, TNF-α, IL-β) and M2 markers (IL-10, Arg-1). The J774.A macrophages, which did not receive any treatment, were labeled as “untreated” group in Fig. 1c. The J774.A macrophages, which received LPS and IFN-γ in order to achieve M1 state, were labeled as “M1 + Panc-1-0h” in the figure, which means the J774A.1 macrophages were treated with LPS and IFN- γ but did not co-culture with Panc-1 cells. The “untreated” group has been used as the control group in comparison of the “M1 + Panc-1-0h” of the experiment. Due to the increase in expression of M1 markers and decrease in M2 marker in “M1 + Panc-1-0h” group compared to the control group, it confirmed that the J774A.1 macrophages were successfully polarized to M1 state after LPS and IFN- γ treatment. Two groups of J774A.1 macrophages (M1 state) were further co-cultured with Panc-1 cells for 48 h and 72 h. The polarity of these two J774A.1 macrophages groups changed to M2-polarized phenotype as indicated by an increase in expression of M2 markers (IL-10, Arg-1) and decrease in M1 markers (iNOS and IL-1 β) compared to M1 control groups (Fig. 1c).

High expression of Arg1 in M2 phenotype and extremely low in M1 phenotype indicated Arg is an optimal M2 specific marker. Similarly, IL-1β and iNOS were chosen as M1 specific markers. The optimal specific marker for M1 and M2 was chosen for future study. In a Transwell co-culture system, the J774A.1 macrophages changed their polarity from M1 to M2 phenotype due to the presence of human pancreatic cancer cells.

### Characterization and Effect of Panc-1 Exosomes on Macrophage Polarization

With the Transwell insert of 3.0 μm pore size in the co-culture system, Panc-1 cells were not able to pass through the insert and directly contact with macrophages. Since there was no direct contact among Panc-1 cells and J774A.1 macrophages, we assumed that there were some “specific intermediary” to deliver the message for macrophage repolarization.

**Figure 2:**
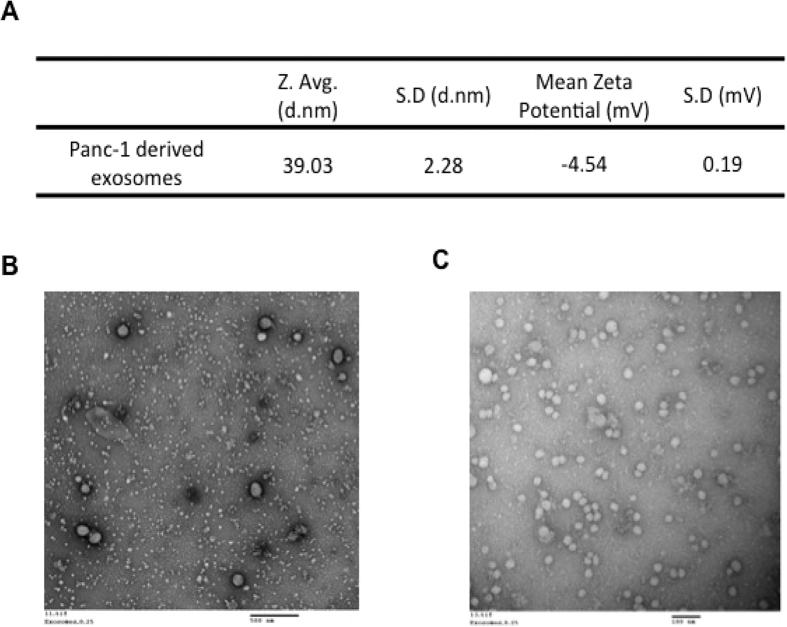
Panc-1 Derived Exosomes Characterization. (**A**) Particle size distribution and zeta potential (surface charge) measurements of Panc-1 exosomes. (**B**) Transmission electron microscopy (TEM) image of exosomes at low magnification. (**C**) TEM image of exosomes at higher magnification.

In our hypothesis, exosomes that secreted by Panc-1 cells might be the key factor for macrophages repolarization. The exosomes that were purified from Panc-1 cells had been characterized by DLS and TEM for their size, charge, and morphology. From the Zetasizer measurement, the average diameter of Panc-1 exosomes was 39 nm, which is between the reported values of 30–100 nm as different cancer cells may secrete exosomes of different sizes^22^. The zeta potential (surface charge) value of −4.54 indicated that Panc-1 derived exosomes negatively charged vehicles (Fig. 2a). TEM showed spherical shape of Panc-1 derived exosomes with size of 30 nm in majority, which was smaller than that measured by DLS and the minority of the exosomes was 100 nm due to aggregation under low magnification of TEM image (Fig. 2b,c).

In order to confirm our hypothesis that exosomes secreted by Panc-1 cells were the key factor for macrophages repolarization, we showed an exosome dose-dependent study of J774A.1 repolarization with Panc-1 exosomes. The isolated and purified exosomes were quantified by their protein concentration via microBCA assay and different protein concentrations of exosomes, 40 μg, 80 μg, 120 μg, 160 μg, were added into the J774A.1 macrophages media for 72 hours. The result indicated that there was a dose-dependent effect on J774A.1 macrophages polarity when dosing the cells with different amount of exosomes. The markers for M1 and M2, which had been optimized from previous studies, were used to determine the phenotype of macrophages. Since polarization of macrophages toward M1 phenotype was associated with an increase in IL-β expression level and a decrease in Arg1 level, a ratio of IL-β/Arg1 was selected as an indication of M1 macrophage polarization. It is clearly that the IL-1β/Arg1 ratio was decreased from 13.76 to 1.44 while the higher protein concentration of Panc-1exosome was given to the macrophages. Dosing 160 ug of exosomes into J774A.1 macrophages presented the IL-1β/Arg1 ratio of 1.44, which is closed to the IL-1β/Arg1 ratio of the Panc-1 cells co-culturing group with the value of 1.14. The study demonstrates that Panc-1 derived exosomes could be one of the factors contributing to J774A.1 macrophages repolarization from M1 to M2 phenotype. (Figure S1).

### Exosome Uptake and Internalization in J774A.1 macrophages

**Figure 3:**
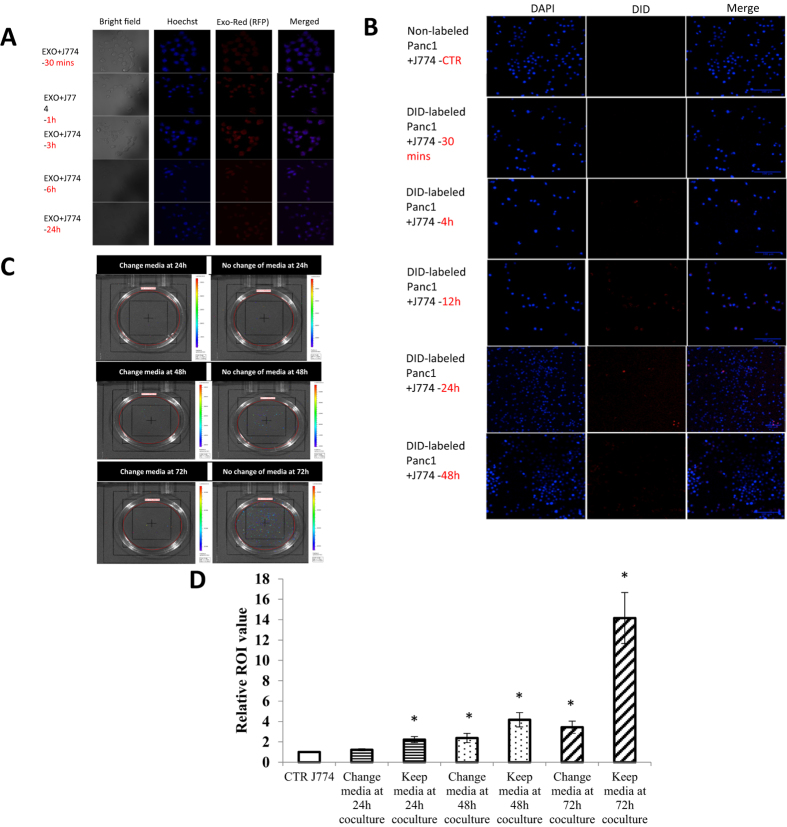
Exosome Uptake Studies in J774A.1 Macrophages. (**A**) Uptake of labeled Panc-1 derived exosomes in J774A.1 macrophages by confocal microscopy. (**B**) Uptake of exosomes from labeled Panc-1 cells in J774A.1 macrophages by confocal microscopy. (**C**) Uptake of exosomes from luciferase-expressing Panc-1 cells in J774A.1 macrophages by IVIS bioluminescence imaging. (**D**) Quantitative analysis of bioluminescence level at 24, 48, and 72 h post-incubation from Transwell co-culture system. n = 3, *p < 0.05 compared to untreated macrophages.

The purpose of following uptake studies were to show the intercellular interaction of exosomes between two different cells in co-culture system and demonstrated how the tumor-derived exosomes be secreted from Panc-1 cells and be taken by J774A.1 macrophages. The first uptake study was carried out by staining exosome RNA with nucleic acid selective fluorescent cationic dye. It is membrane permeable and can fluorescently label single-stranded RNAs inside of exosomes by electrostatic attractions. We investigated cellular uptake of these labeled exosomes in J774A.1 macrophages by confocal microscopy. The image showed that red fluorescent signal from labeled exosomes was observed in J774A.1 cell at 30 min of incubation. Increasing incubation time to 1 h and 3 h increased the signal intensity. Bright signal of labeled exosomes was observed in the cells after 3 h of incubation. However, the signal intensity did not increase when increase incubation time to 6 h and 24 h (Fig. 3a). The fluorescence shown in cells suggested that the labeled exosomes were effectively internalized by J77A.1 macrophages. Labeling the exosomes and dosing these exosomes into J774A.1 cells provided a direct method of exosomes uptake studies. Since the labeled exosomes were incubated with J774A.1 macrophages through culture media, the signal started to present in J774A.1 macropahges after 30 minutes incubation, and increased the signal intensity by 3 hours incubation. J774A.1 macrophages could engulf the exosomes presented in the media and digested these labeled exosomes shortly, resulting in the signal decreased at 6 h and 24 h incubation time points.

The second uptake study conducted with the aim of developing an exosomes-tracking model in Panc-1 cells and macrophage indirect co-culture system. Panc-1 cells were labeled by stable DiD dye on the membrane and were seeded into the upper chamber of the Transwell insert. Since exosomes are endosome-derived intraluminal vesicles that were secreted by Panc-1 cells, we hypothesized that these exosomes would fuse with the plasma membrane and carry a piece of labeled membrane from Panc-1 cells when releasing into the media and be internalized by J774A.1 macrophages at the bottom of the co-culture system. We further investigated cellular uptake of these exosomes in J774A.1 macrophages by confocal microscopy and showed that red fluorescent signal (DiD) from labeled Panc-1 cells was observed in J774A.1 macrophages starting from 4 hour of co-culture incubation. Increasing incubation time to 12 hours and 24 hours presented the highest signal intensity. However, the signal intensity decreased at 48 hours and 72 hours incubation time (Fig. 3b). Although the overall signal intensity was low, the exosomes uptake study here provides a solid demonstration on exosomes secretion and uptake in J774A.1 macrophages.

Since it took time for the exosomes to fuse with the panc-1 plasma membrane, carry a piece of labeled membrane from panc-1 cells, and release into the media and be internalized by J774A.1 macrophages, the earliest flurescence signal showed in J774A.1 appeared much later (4 h) than the first direct study (30 mins). Due to the design of the study, the amount of exosomes need to be large enough for the fluorescence signal on their membrane to be observed under the microscope, hence, the maximum signal intensity was shown at 24 hours and started to decrease at 48 and 72 hours. Due to the direct treatment of the labeled exosoms, the maximum signal intensity presented at an earlier time point (3 h) comparing to the indirect exosomes uptake study, which showed the maximum signal intensity at 24 h. The more completed and longer process for panc-1 cells to secret their exosomes and release into the microphages co-culture system results in the discrepancy of the time line for signal intensity in the two uptake studies.

In the third uptake study, luciferase-expressing Panc-1 cells (Panc-1 luc) were used instead of regular Panc-1 cells in order to study the cellular uptake and intracellular interaction of exosomes in indirect co-culture system via luciferase activity. *In vitro* bioluminescence activity of Panc-1 luc cells was confirmed by an *in vitro* activity assay and evaluated as a function of number of cells to ascertain that the signal response increases with the increase in the total number of cells. Panc-1-luc showed a positive correlation between the luminescence intensity signal and the number of cells (data not shown). The uptake studies of Panc-1 luc cells and J774A.1 macrophages co-culture system were evaluated at 24 hour, 48 hour, and 72 hour post-incubation. Only the bottom part of the co-culture system, which contains J7741.A macrophages, was imaged under lumina II *in vivo* imaging system for bioluminescence signal determination. The IVIS images proved that the uptake increased as the time progressed and maximum uptake as evident from bioluminescence signal was observed at the 72 h post-incubation. We investigated each time point with two different conditions. After removing the Transwell insert on the top, the macrophage media were either removed or maintained before imaging. The media retained at each time point resulted in lower bioluminescence signal compared to media removal group, suggesting that the exosomes secreted from Panc-1 luc cells with bioluminescence signal were presented in the media, and increased as the time progressed. It was concluded that the exosomes secreted by Panc-1 luc cells were presented more in culture media and less were internalized by J774A.1 macrophages (Fig. 3c). The ROI value of each condition at each time point was shown as an indicator of exosomes uptake in J774A.1 macrophages (Fig. 3d). The regular Panc-1 cells co-cultured with J774A.1 macrophages were carried out as a control group and its ROI value can be seen as the IVIS imaging background signal. This study provided crucial evidence in the presence of exosomes in media, and these exosomes were accumulated over time. In these exosomes uptake studies, we obtained a comprehensive picture and confirm that tumor-derived exosomes secreted from cancer cells could be internalized by J774A.1 macrophages.

### HA-PEI/HA-PEG Nanoparticles-Encapsulated miR-155 and miR-125b Expressing Plasmid DNA and Transfection Studies in Panc-1 Cells

**Figure 4:**
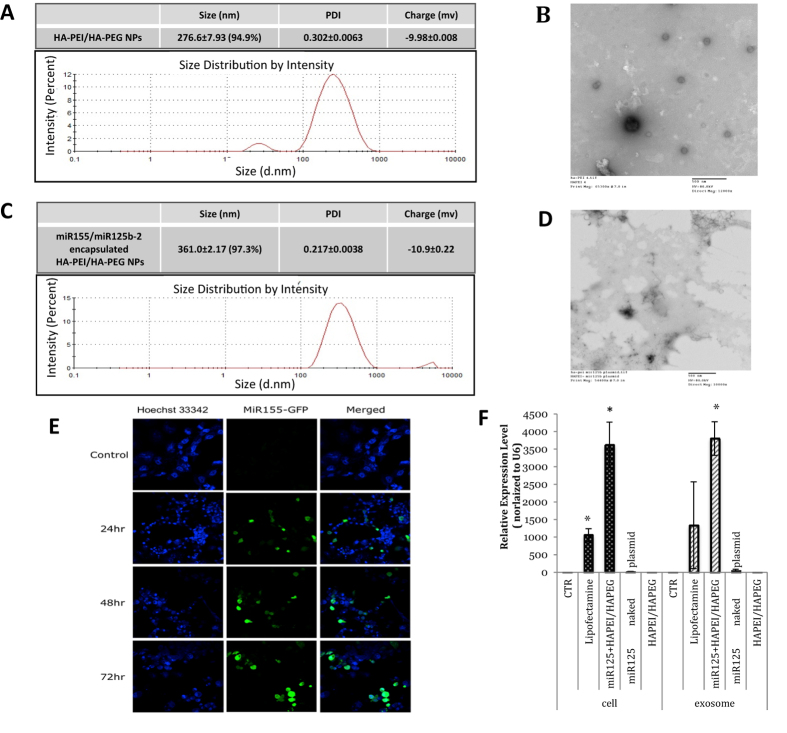
Characterization of HA-PEI/HA-PEG Nanoparticles and Transfection of miR-155 and miR-125b in Panc-1 Cells using miR-Expressing Plasmid DNA. (**A**) HA-PEI/HA-PEG nanoparticle size distribution and zeta potential (surface charge) measurements of blank HA-PEI/HA-PEG in PBS by dynamic light scattering. (**B**) Transmission electron microscopy (TEM) image of blank HA-PEI/HA-PEG in PBS. (**C**) Size distribution and zeta potential measurements of miR125b-2 encapsulated HA-PEI/HA-PEG in PBS (9:1) by dynamic light scattering. D. TEM image of miR-125b-2 encapsulated HA-PEI/HA-PEG in PBS (9:1). (**E**) Fluorescent confocal microscopy images of miR-155/GFP plasmid DNA expression in Panc-1 cells at 12 h, 24 h, and 48 h post-transfection. (**F**) Quantitative analysis of miR125b-2 plasmid DNA transfection studies in Panc-1 cells and exosomes upon transfection with Lipofectamine^®^ and HA-PEI/HA-PEG nanoparticles. pPCR analysis was performed by miR125b-2 Taqman gene expression assay with specific miR-125b-2 Taqman primer. n = 3, *p < 0.05 compared to non-transfected Panc-1 cells and Panc-1 derived exosomes.

HA-PEI could form a complex with miRNA plasmid DNA due to the electrostatic interaction between positively charged PEI and negatively charged plasmid DNA. The particle size and zeta potentials of blank and miR125b-2 encapsulated HA-PEI/HA-PEG self-assemble nanoparticles were examined using the NanoZetasizer^®^ 90 instrument. The instrument measures the size of the particle based on the principle of dynamic light scattering (DLS) and the zeta potential based on the principle of electrophoretic mobility of the particle. The mean Z-average particle size of HA-PEI/HA-PEG nanoparticles was 276.6 ± 7.93 nm (Fig. 4a) and the particle size of miR125b-2 encapsulated HA-PEI/HA-PEG was 361 ± 2.17 nm, which was increased after miRNA plasmid DNA encapsulation (Fig. 4c). As shown in Fig. 4a,c, a negative surface charge was presented by both HA-PEI/HA-PEG and miR125b-2 encapsulated HA-PEI/HA-PEG nanoparticles. The zeta potential of blank nanoparticles was −9.98 ± 0.008 mV and similarly, the zeta potential of miR125b-2 encapsulated nanoparticles was −10.9 ± 0.22 mV due to the contribution of both PEG and HA which are negatively charged. The surface morphology of both the nanoparticles was found to be spherical under TEM. It showed spherical shape of HA-PEI/HA-PEG with size in the range of 100–223 nm with a few of larger nanoparticles due to aggregation, which was smaller than that measured by DLS (Fig. 4b). TEM image suggested a dark core for miR125b-2 encapsulated nanoparticles, which may be attributed to the high contrast arising from the uranyl acetate stained miR125b-2 plasmid DNA loaded in the core of the nanoparticles (Fig. 4d). The size of miR125b-2 encapsulated nanoparticles under TEM was in the range of 150–250 nm which was also smaller than that measured by DLS.

The plasmid DNA-encapsulated nanoparticles were then run on 0.8% Agarose E-gel to check percent encapsulation of the plasmid. Plasmid DNA encapsulation was further confirmed by decomplexing HA-PEI/HA-PEG miR125b-2 plasmid DNA with anionic poly(acrylic acid) (PAA) by mixing an equal volume of HA-PEI/HA-PEG-plasmid DNA and 2% PAA using a vortex mixer. The strongly anionic PAA displaces the plasmid by electrostatically interacting with the cationic PEI. The decomplexed samples were further run on a 0.8% Agarose E-gel to ensure the presence of intact plasmid bands. The result showed that the naked plasmid DNA did not degrade and the band appeared at 5,000 b.p. as indicated by 1kb DNA ladder. The plasmid DNA encapsulated nanoparticles was no band presented, indicated that the plasmid had been encapsulated in the nanoparticles. The 1:9 ratio of plasmid: HA-PEI/HA-PEG was optimized by this study. After treating the encapsulated plasmid with 2% PAA to rupture the nanoparticles, the plasmid released and was observed on the gel (Figure S2).

The ability of HA-PEI/HA-PEG nanoparticles to carry and efficiently deliver miR155 plasmid DNA was evaluated by *in vitro* transfection of HA-PEI/plasmid DNA nanoparticles in Panc-1 cells. After eliminating the auto-fluorescence of control Panc-1 cells, the cells that had been successfully transfected showed the GFP expression and the green signal increased from 24 h, 48 h, to 72 h transfection time period (Fig. 4e). The result suggested that the plasmid DNA had been transfected into cells and it could be used for a long-term delivery as the plasmid DNA was steadily expressing. qPCR was used for quantitative determination of miR125b-2 gene expression levels in Panc-1 cells and Panc-1 derived exosomes after transfection with HA-PEI/HA-PEG plasmid DNA, Lipofectamine^®^/plasmid DNA, naked plasmid, and blank HA-PEI/HA-PEG at 48 h post-transfection. HA-PEI/HA-PEG plasmid DNA nanoparticles mediated transfection significantly increased miR125b-2 expression up to 3600 fold in Panc-1 cells and 3100 fold in Panc-1 derived exosomes 48 h transfection. The expression level of miR125b-2 in Panc-1 cells and exosomes transfected with Lipofectamine^®^/plasmid DNA was about 1000 and 1300 fold higher than that in untreated cells at 48 h, respectively (Fig. 4f). Our study showed that transfection levels with HA-PEI/HA-PEG plasmid DNA were higher than those with Lipofectamine®/plasmid DNA at 48 h. It indicated that HA-PEI/HA-PEG nanoparticles was more effective in maintaining high transgene expression up to 48 h compared to Lipofectamine®, a widely used commercial transfection reagent. We concluded that the HA-PEI/HA-PEG nanoparticles formulation was a suitable carrier for plasmid DNA delivery and was capable of delivering miR125b-2 plasmid DNA to the cells and modifying the cargo in exosomes with the highest efficacy.

### Panc-1 Exosome Composition Analysis Following Cellular Transfection with miR-155 and miR-125b Expressing Plasmid DNA

**Figure 5:**
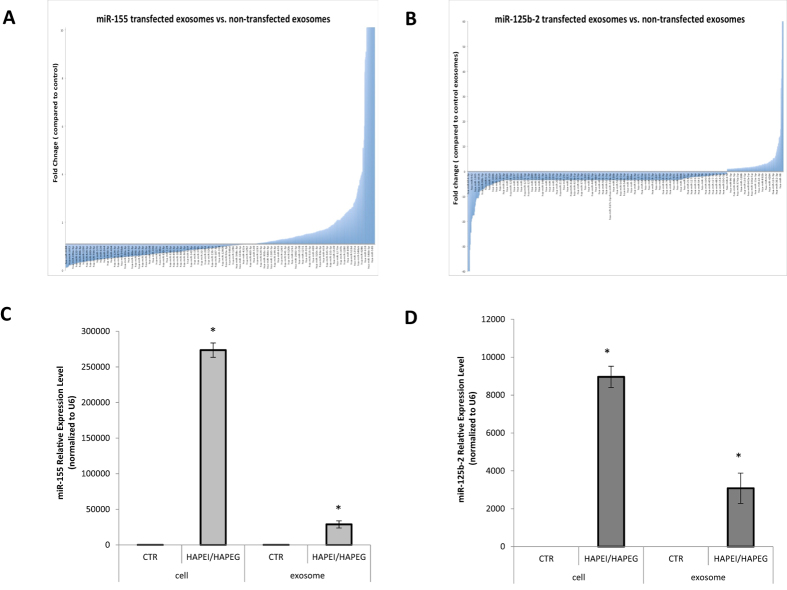
Nanostring Microarray Analysis of Changes in microRNA Content in the Panc-1 Exosomes after Transfection with miR-155 and miR-125b Expressing Plasmid DNA. (**A**) Relative fold change as shown in the water-fall plot in microRNAs composition from Panc-1 exosomes after miR-155 transfection as compared to non-transfected exosomes. (**B**) Relative fold change in microRNAs composition from Panc-1 exosomes after miR-125b-2 transfection as compared to non-transfected exosomes. (**C**) The expression level of miR-155 transcript in miR-155 plasmid DNA transfected Panc-1 cells and exosomes by specific miR-155 Taqman primer (**D**) The expression level of miR-125b-2 transcript in miR-125b-2 plasmid DNA transfected Panc-1 cells and exosomes by specific miR-125b-2 Taqman primer. n = 3, *p < 0.05 compared to non-transfected Panc-1 cells and Panc-1 derived exosomes.

The miR-155/miR-125b-2 modified exosomes secreted from Panc-1 cell were collected after transfection and the microRNAs from these exosomes were extracted purified for exosomes microRNA microarray analysis at Dana-Farber Cancer Institute. The purpose of the study is to present a comprehensive microRNA profiling in order to understand the change of microRNA cargo in exosomes after transfection. The exosomes profiling data was performed by using nSolver analysis software for miR-155 plasmid DNA transfected exosomes, miR-125b-2 plasmid DNA transfected exosomes, and non-transfected exosomes microRNAs normalization. The ratio of each microRNA expression level in miR-155 and miR-125b-2 transfected exosomes compared to non-transfected exosomes was shown in waterfall plot (Fig. 5a,b). This study showed the different microRNAs from Panc-1 derived exosomes had different fold changes, indicating that these microRNAs had been either upreguated or downregupated after miR-155/miR-125b-2 plasmid DNA transfected compared to non-transfected exosomes. With the same transfection protocol, we also investigated the miR-155 and miR-125b expression level in miR-155 plasmid DNA and miR-125b plasmid DNA transfected Panc-1 cells and exosomes by taqman qPCR assay with specific miR-155 and miR-125b primers. The transfected cells showed a significant increase of miR-155 gene expression level in Panc-1 cells with 2.7 × 10^5^ fold increase and in exosomes with 2.8 × 10^4^ fold increase after miR-155 plasmid DNA transfection compared to control (Fig. 5c). In contrast, a 8900 fold increase of miR-125b-2 expression in cells and a 3000 fold increase of miR-125b-2 expression in exosomes were shown after miR-125b-2 plasmid DNA transfection (Fig. 5d). Our study suggested that there was a significant increase of miR-155 and miR125b-2 gene expression level in both Panc-1 cells and exosomes after miR-155/miR-125b-2 plasmid DNA transfection. The high expression level of these two microRNAs in our modified exosomes might contribute to microphage reprogramming, which possess the function for M2 phenotypes macrophage rehabilitate back to M1 phenotype.

### Reprogramming Macrophages with Harvested Exosomes from Transfected Panc-1 Cells

**Figure 6:**
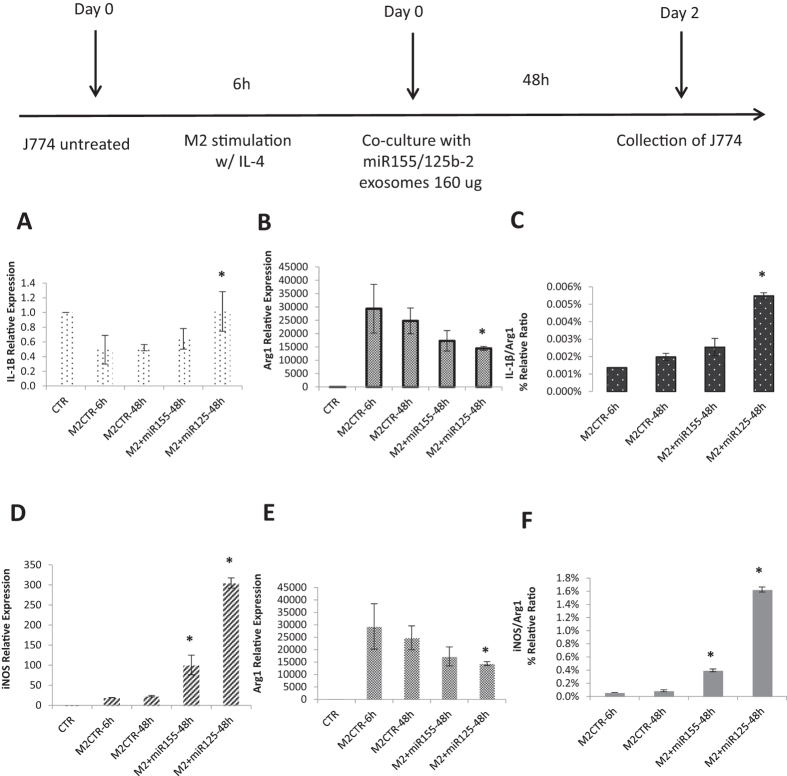
Macrophage Re-programming with Exosomes Harvested from miR-155 and miR-125b-2 Transfected in Panc-1 Cells. Quantitative determination of (**A**) IL-1β and (**B**) Arg1 expression in M2 J774A.1 macrophages after treating 160 μg of miR-155/miR-125b-2-modified exosomes for 48 h. (**C**) IL-1β/Arg1 (M1/M2) ratio of M2 J774A.1 macrophages treated with miR-155/miR-125b-2-modified exosomes for 48 h. Quantitative determination of (**D**) iNOS and (**E**) Arg1 expression in M2 J774A.1 macrophages after treating 160 μg of miR-155/miR-125b-2-modified exosomes for 48 h. (**F**) iNOS/Arg1 (M1/M2) ratio of M2 J774A.1 macrophages treated with miR-155/miR-125b-2-modified exosomes for 48 h. n = 3, *p < 0.05 compared to M2 macrophage treated with IL-4 for 6 h and keep for 48 h.

It was confirmed by our previous study that the Panc-1 derived exosomes were able to trigger the macrophage re-polarization from M1 phenotype to M2 phenotype. Thus, change of Panc-1 derived exosomes cargo contained highly miR-155 and miR-125b-2 expression to achieve macrophage re-programming effect from M2 back to M1 phenotype was investigated in this study. The modified exosomes treated experimental groups were carried out dosing 160 μg of miR155 or miR125b-2 modified exosomes in M2 phenotype macrophages for 48 hours after 6 h IL-4 treatment. J774A.1 macrophages treated with IL-4 for 6 hours and kept for 48 hours were conducted as M2 phenotype controls. The miR155/miR125b-2 expression level in J774A.1 macrophages was firstly be investigated by Taqman qPCR assay with specific miR-155 and miR-125b-2 Taqman primer. The result showed that the levels of miR155 and miR125b-2 in J774A.1 macrophages increased 2.22 and 11.5 fold respectively 48 h post treatment compared to control groups (Figure S3). To investigate if high expression of miR-155 and miR-125b-2 in exosomes could re-polarize the macrophages from M2 to M1 phenotypes, qPCR was used to measure IL-1β/Arg1 (Fig. 6a–c) and iNOS/Arg1 ratio (Fig. 6d–f) in the M2 phenotype treated with miR-155 or miR-125b-2 modified exosomes for 48 h. Due to high expression of iNOS, IL-1β and low expression of Arg1 in M1 macrophages, the high iNOS/Arg1 and IL-1β/Arg1 ratio was used to represent the majority of macrophage in M1 phenotype. This ratio was slightly increased in miR-155 modified exosomes treated group and significantly increased in miR-125b-2 modified exosomes treated group. The results indicated that J774A.1 macrophages were effectively re-polarized from M2 to M1 state in the presence of either 160 μg of miR155 or miR125b-2 modified exosomes for 48 h comparing to the IL-4 treated M2 phenotype macrophages control group.

### Reprogramming Macrophages with Exosomes from Transfected Panc-1 Cells in the Transwell Co—Culture System

**Figure 7:**
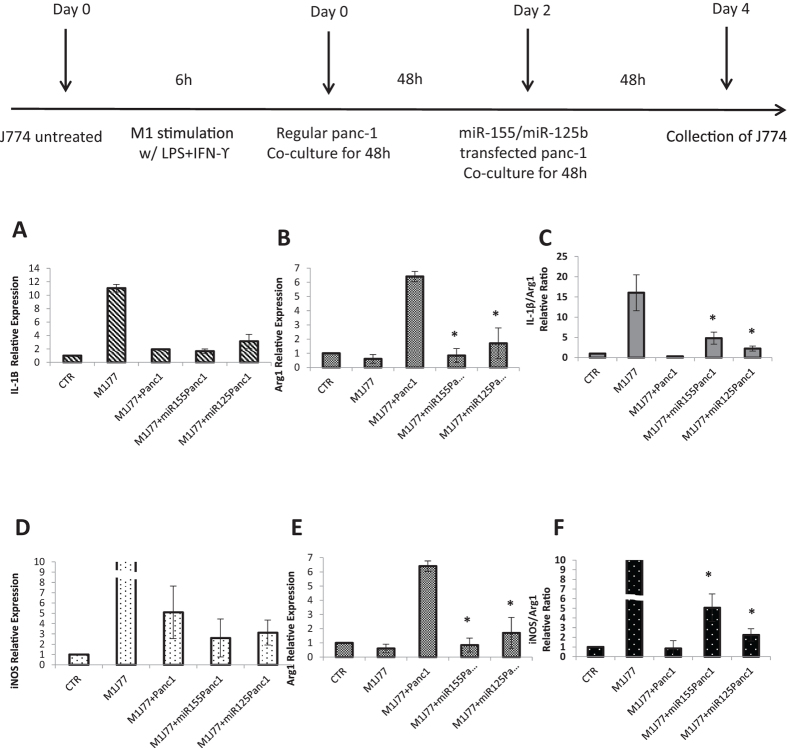
Macrophage Reprogramming with Exosomes Transferred in Transwell Co-Culture System from miR-155 and miR-125b-2 Transfected Panc-1 Cells. Quantitative determination of (**A**) IL-1β and (**B**) Arg1 expression in M2 J774A.1 macrophages after co-culturing with miR-155/miR-125b-2-modified transfected panc-1 cells for 48 h. (**C**) IL-1β/Arg1 (M1/M2) ratio of M2 J774A.1 macrophages co-culturing with miR-155/miR-125b-2-modified transfected panc-1 cells for 48 h. Quantitative Determination of (**D**) iNOS and (**E**) Arg1 expression in M2 J774A.1 macrophages co-culturing with miR-155/miR-125b-2-modified transfected panc-1 cells for 48 h. (**F**) iNOS/Arg1 (M1/M2) ratio of M2 J774A.1 macrophages co-culturing with miR-155/miR-125b-2-modified transfected panc-1 cells for 48 h. n = 3, *p < 0.05 compared to M1 macrophage co-culture with regular panc-1 cells group.

After the macrophages re-programming effect of microRNA-modified exosomes on J774A.1 macrophages, a further study was carried out using miR155/miR125b-2 plasmid DNA transfected Panc-1 cells in indirect co-culture system to assess the ability of macrophages re-programming effect from the exosomes secreted by these transfected Panc-1 cells. Based on the same protocol for Panc-1 cells and J774A.1 macrophages in Transwell co-culture system, untreated J774A.1 macrophages were stimulated with IFN-γ and LPS for 6 h to induce their polarization to M1 phenotype. The Transwell inserts containing regular Panc-1 cells were placed into the culture dishes containing J774A.1 macrophages, and incubated up to 48 hours to enable the macrophages polarize to M2 state. The miR155/miR125b-2 plasmid DNA transfected Panc-1 cells were seeded on new Transwell inserts by same transfection protocol in previous studies. The Transwell inserts were then used to replace the inserts containing regular Panc-1 cells in co-culture system for 48 hours. The effect of miR155/miR125b plasmid DNA transfected Panc-1 cells on macrophages polarity was determined by using qPCR. Our result revealed that the expression of Arg1 decreased significantly and the expression of IL-1β increase slightly compared to regular Panc-1 cells co-culture group after co-culturing with miR-155 and miR-125b-2 plasmid DNA transfected Panc-1 cells (Fig. 7a,b). It could be clearly seen that IL-1β/Arg1 ratio increased in both miR-155 and miR-125b-2 plasmid DNA transfected Panc-1 cells co-culture groups. However, the expression level of another M1 marker iNOS in miR-155/miR-125b-2 plasmid DNA transfected Panc-1 cells co-culture groups also decreased in comparison to control co-culture group (Fig. 7d). We again determined the macrophage polarity based on its iNOS/arg1 ratio (Fig. 7f). After normalizing with their own arg1 value, the iNOS/arg1 ratio of miR-155 and miR-125b-2 transfected Panc-1 cells co-culture group both increased compare to control co-culture group. The increase of IL-1β/Arg1 and iNOS/Arg1 ratio both confirmed that modified exosomes secreted by miR155 and miR125b-2 plasmid DNA transfected Panc-1cells contribute to the macrophage re-programming from M2 phenotype back to M1 phenotype.

## Discussion

In cancer, signals via exosomes affect the immune system by inducing alternative activation of macrophages. Macrophages could be polarized to different phenotypic states depending on the type of stimuli used. J77A.1 macrophages were polarized to M1 phenotype by treatment with 100ng/ml LPS/IFN-γ for 6 hours and to M2 phenotype using 100ng/ml IL-4 for 6 hours thus demonstrating their plastic nature. The phenotype was indicated by the change in M1 and M2 specific markers. We developed an *in vitro* transwell co-culture system to demonstrate how the J774A.1 macrophages re-polarize their phenotype from classically activated macrophages (M1) to alternatively activated macrophages (M2) due to the presence of human pancreatic cancer cells. Since there was no direct contact among Panc-1 cells and J774A.1 macrophages, we assumed that there were some “specific intermediaries” to deliver the messages for cell-cell communication. Accumulating evidence reveals that the exosomes secreted by various cell types might play an important role in transmitting the messages via their microRNA, mRNA, or protein cargo. The exosomes that were purified from Panc-1 cells had been characterized, which were around 40 nm in size and the zeta potential was −4.54 ± 0.19 mV. Dosing the tumor-derived exosomes into J774A.1 macrophages showed the same trend when co-cultured Panc-1 cells with J774A.1 macrophages, which demonstrates that Panc-1 derived exosomes could be one of the factors contributing to J774A.1 macrophages re-programming from M1 to M2 phenotypes.

Cellular uptake studies using labeled exosomes and labeled Panc-1 cells revealed the intercellular tracking of exosomes in co-culture system and demonstrated how the tumor-derived exosomes be secreted from cancer cells and be taken by the recipient cells. In addition, panc-1 Luc cells co-cultured with J774A.1 macrophages in bioluminescence imaging confirmed the presence of exosomes cumulated in the media over time.

Next, microRNA 155 and microRNA 125b-2 encoding plasmid were encapsulated in the HA-PEI/HA-PEG nanoparticles and the average size of the resulting nanoparticles was 361 nm, which is slightly larger than blank HA-PEI/HA-PEG nanoparticles with the average size of 276.6 nm. The zeta potential of both blank and loaded nanoparticles was negative due to the contribution of PEG and HA which are negatively charged. The encapsulation study done using E-gel confirmed the microRNAs expressing plasmid DNA was completely encapsulated in the nanoparticles without leakage.

The miR-155/miR-125b-2 plasmid DNA encapsulated HA-PEI/HA-PEG nanoparticles were used in panc-1 cells transfection and evaluate the transfection efficacy by comparing to Lipofectamine. The result indicates that HA-PEI/HA-PEG formulation was better than Lipofectamine in causing gene transfection. Therefore, non-viral HA-PEI/HA-PEG delivery system was further applied for gene therapy in this project. The transfected panc-1 cells and panc-1 derived exosomes both showed very high microRNA expression level comparing to non-transfected cells and exosomes. The miR-155/miR-125b-2 modified exosomes were also being processed for a comprehensive microRNA profiling study in order to understand the change of microRNA cargo in exosomes after miR-155/miR-125b-2 plasmid DNA transfection.

After verifying the role of exosomes in switching J774A.1 macrophage polarization state from M1 to M2 phenotype, a further study of J774A.1 macrophage re-programming from M2 back to M1 state was carried out using the microRNA modified panc-1 cells in indirect co-culture system or dosing 160ug of modified exosomes into J774A.1 macrophages. The increase of iNOS/Arginase or IL-1β/Arginase ratio in J774A.1 macrophages suggested that M2 phenotypes J774A.1 macrophages were rehabilitated back to M1 phenotype due to the presence of miR-155/miR-125b-2 modified exosomes.

In conclusion, our results demonstrate that modifying the cargo inside the exosomes by hyaluronic acid-poly(ethylene imine)-based nanoparticle delivery system (HA-PEI/HA-PEG) encapsulated plasmid DNA expressing miR-155 or miR-125b-2 can achieve stable expression of the microRNAs, and these modified tumor-derived exosomes can result in macrophages reprogramming in pancreatic tumor microenvironment. After understanding the possible mechanism to recruit more TAMs as M2 phenotypes, which could already been shown the effect in cancer progression, the non-viral delivery system was conducted to deliver specific microRNA-155 and microRNA-125b, which possess the function for M2 phenotypes macrophage rehabilitate back to M1 phenotype. The successfully reprogramming of TAMs might hold a potential against tumor invasion and metastasis in pancreatic cancer pathogenesis.

## Methods

### Materials

Sodium hyaluronate (HA) with an average molecular weight of 20,000 Da was purchased from Lifecore Biomedical Co. (Chaska, MN). Branched poly (ethyleneimine) (bPEI) with an average molecular weight of 10,000 Da was purchased from Polysciences Inc. (Warrington, PA). Mono-functional PEG amine (mPEG2k- NH2, MW 2 kDa) was purchased from Creative PEG Works (Winston Salem, NC). N- (3-dimethylaminopropyl)-N’-ethylcarbodiimide hydrochloride (EDC), N-hydroxysuccinimide (NHS), and lipopolysaccharide (LPS) were purchased from Sigma-Aldrich Chemical Co. (St. Louis, MO, USA). Recombinant interferon-gamma (IFN-γ, murine interleukin-4 (IL-4), and interleukin-10 (IL-10) were obtained from PeproTech (Rocky Hill, NJ). Platinum TaqDNA^®^ Polymerase, Total Exosome RNA & Protein Isolation Kit, 4′, 6-diamidino-2-phenylindole (DAPI) were purchased from Invitrogen (San Diego, CA). Verso cDNA Synthesis Kit was purchased from Thermo Scientific (Pittsburgh, PA). LightCycler 480 SYBR Green I Master Mix and High Pure RNA Isolation kit were purchased from Roche Applied Sc. (Indianapolis, IN). Plasmid Giga Kits were obtained from Qiagen (Valencia, CA). 2% E-gels with SYBR Safe and 1% EX E-gels, Lipofectamine 3000, TaqMan MicroRNA Reverse Transcription Kit, and nuclease-free water were purchased from Life Technologies (Woburn, MA). Exo-FBS Exosome-depleted FBS, ExoQuick-TC Exosome Precipitation Solution, and Exo-Red exosome RNA fluorescent label solution were purchased from System Biosciences (Mountain View, CA). CellBrite Red Cytoplasmic Membrane DiD Dye was purchased from Biotium (Hayward, CA). miRNASelect pEGP-mmu-miR-125b-2 and pEGP-mmu-mir-155 Expression Vector were purchased from Cell BioLabs (San Diego, CA). Murine primers specific for TNF-α, iNOS-2, IL-1β, Arg-1, IL-4, IL-10 and β-actin were purchased from Eurofins MWG Operon (Huntsville, AL).

### Cell Culture Experiments

J774A.1 adherent murine macrophage cell line and human pancreatic ductal adenocarcinoma cell line Panc-1 were obtained from American Type Culture Collections (ATCC, Manssas, VA), while luciferase-expressing Panc-1 cell line (Panc-1 luc) was kindly provided by Prof. Dawn E. Quelle from the Carver College of Medicine, University of Iowa (Iowa City, IA). All the cell lines were subcultured in Dulbecco’s Modified Eagle Medium (DMEM) (Cellgro, Manassas, VA) supplemented with 10% Fetal Bovine Serum (FBS) (HyClone, Logan, UT) and 1% penicillin/streptomycin antibiotics (Gibco Invitrogen, Woburn, MA) at 37 °C and 5% CO_2_.

### Panc-1 Exosomes Isolation and Purification

Exosomes were isolated from the supernatant of Panc-1 cell culture media. 2 × 10^6^ cells were plated in T175 flasks in DMEM supplemented with 10% FBS and 1% penicillin/streptomycin. The following day, media were changed to exosome-free DMEM supplemented with 10% Exosome-depleted FBS. The culture media were collected and isolated by following the manufacturer protocol. After isolation, exosomes were re-suspended in 1X PBS and stored at −20 °C until further use.

The morphology and the size of the exosomes were imaged by Transmission Electron Microscopy (TEM). Specimens were prepared by adding a suspension of the exosomes or nanoparticles dropwise to a formvar/carbon film grid followed by air-drying, and stained with negative uranyl acetate dye for imaging.

### Panc-1 Exosome Uptake in J774.A1 Macrophages

In order to characterize exosomes uptake and internalization in J774A.1 macrophages, three different exosomes uptake studies were carried out using confocal microscopy and IVIS bioluminescence imaging to study the role of exosomes on communicating and transmitting information between host cells and recipient cells comprehensively.

For the direct Exosomes uptake studies, exosomes isolated from Panc-1 cells were stained with Exo-Red exosome RNA fluorescent label solution by following the manufacturer protocol. A total of 2 × 10^5^ J774A.1 cells were grown in a 6-well plate in DMDM. Labeled exosomes (100 μl) was added into each well for 30 min, 60 min, 180 min, 360 min, and 24 hours of incubation. Then the cells were washed with 1X PBS twice. Nuclear was stained with 5 μl/ml of Hoechst 33342 for 15 min. The cells were fixed with 4% w/v formaldehyde for 10 min. A drop of prolong anti-fade mounting media was placed on a slide and the coverslip in each well was picked up with forceps and placed on the slide. Then the slides were viewed under a Zeiss LSM 700 scanning confocal microscope and observed at 460 nm excitation and 650 nm emission wavelength under the microscope.

For indirect exosomes uptake studies, Panc-1 cells were labeled by CellBrite Red Cytoplasmic Membrane DiD Dye following the manufacturer protocol. A total of 1 × 10^6^ DiD labeled Panc-1 cells were seeded into the upper chamber of a 75 mm Polycarbonate Transwell insert of 3.0 μm pore size in DMEM media. A total of 1.2 × 10^6^ of J774A.1 macrophages were seeded in 100 mm polystyrene cell culture dishes in DMEM media. The following day, the culture inserts with DiD labeled Panc-1 cells were placed into the 100 mm cell culture dishes containing J774A.1 macrophages. The J774A.1 macrophages were collected at multiple time periods after incubation at 37 °C. The cells were washed with 1X PBS twice, and were fixed with 4% w/v formaldehyde for 10 min. A drop of DAPI Fluoromount-G^®^ was placed on each slide. The slides were made with the same protocol and viewed under Zeiss LSM 700 scanning confocal microscope. Cy5.5 (647 nm filter) was used for J774.1 macrophages and DAPI for nuclear stain.

Another indirect exosomes uptake studies were carried out using Luciferase-expressing Panc-1 cells (Panc-1 luc) instead of regular Panc-1 cells in indirect co-culture system. A total of 1 × 10^6^ Panc-1 luc cells were seeded into the upper chamber of a 75 mm Transwell insert of 3.0 μm pore size in DMEM media and a total of 1 × 10^6^ of J774A.1 macrophages were seeded in 100 mm polystyrene cell culture dishes in DMEM media. The following day, the culture inserts with Panc-1 luc cells were placed into the 100 mm cell culture dishes containing J774A.1 macrophages. The J774A.1 macrophages were collected at 24 hours, 48 hours, and 72 hours after incubation at 37 °C. 10 ul of 15 mg/ml D-Luciferin was dissolved in ice-cold PBS, and added into each 100 mm cell culture dish containing 10ml DMEM media. Then the dish was imaged under Lumina II *in vivo* imaging system (IVIS) from Caliper Life Sciences (Hopkinton, MA).

### Amplification and Purification of miR-155 and miR-125b Expressing Plasmid DNA

The microRNA constructs miRNASelect pEGP-mmu-mir-155 and pEGP-mmu-miR-125b-2 Expression Vector were provided as bacterial glycerol stock in transformed *E. coli*. Inoculation of 5 ml of LB media with a loop of frozen bacterial glycerol stock was been prepared by incubating at 37 °C for 12 hours with shaking at 250 rpm. The plasmid transformed in bacteria suspension was streaked onto LB Agar Ampicillin plate using a sterile inoculation loop followed by incubating the plate at 37 °C for 16 hours. Five colonies were then picked into 15 ml of LB Ampicillin Media for a starter culture. The media were incubated at 37 °C for 16 hours with shaking at 250 rpm. The starter culture was diluted at 1:500 ratio in LB Ampicillin Media and incubated at 37 °C for 16 hours with shaking at 250 rpm. Plasmids were then harvested and purified using a QIAFilter Giga Kit by following the manufacturer’s instructions. The plasmid purity was confirmed by measuring the absorbance ratio at 260/280 nm.

### Synthesis, Preparation and Characterization of Plasmid DNA-Containing Nanoparticles

HA-PEI and HA-PEG were synthesized as previous described with minor modification^44^,^49^. HA was chemically conjugated with PEI by using EDC and sulfo-NHS. For the preparation of the PEGlyate hyaluronic acid, HA was chemically conjugated with PEG2000-NH2 using EDC and sulfo-NHS^48^. To prepare HA-PEI/HA-PEG nanoparticles loaded with miR-155 and miR-125b-2 plasmid DNA, a mixture of HA-PEI and HA-PEG were prepared by dissolving 3 mg of HA-PEI conjugate and 3 mg of HA-PEG conjugate in 1 ml of sterile phosphate buffer saline (PBS, pH 7.4). 450 μg of HA-PEG and HA-PEI were mixed with 100 μg of plasmid (miR-155, miR-125b-2) to achieve weight ratio of 9:1 for HA-PEI/HA-PEG and plasmid DNA. HA-PEG was mixed with plasmid DNA, followed by adding HA-PEI while vortexing, and sonicated at room temperature for 30 minutes.

A 0.8% Agarose E-gel electrophoresis was performed to determine percent encapsulation of the miR-155/miR-125b-2 expressing plasmid DNA. Solutions of HA-PEI/HA-PEG nanoparticles containing miR-155 and miR-125b-2 plasmid were made of plasmid: HA-PEI/HA-PEG in 1:9 ratio (%v/v). Plasmid encapsulation was further confirmed by decomplexing encapsulated nanoparticles with anionic poly(acrylic acid) (PAA) by mixing an equal volume of HA-PEI/HA-PEG-miRNA plasmid DNA and PAA using a vortex mixer. The strongly anionic PAA displaces the plasmid by electrostatically interacting with the cationic PEI. The decomplexed samples were then run on the E-gel to ensure the presence of intact plasmid bands. The 1 KB ladder was used as the marker of the gel.

The average particle size, size distribution and zeta-potential of HA-PEI/HA-PEG nanoparticles, HA-PEI/HA-PEG miRNA plasmid DNA nanoparticles were measured using a dynamic light scattering (DLS) instrument NanoZetasizer^®^ 90. Mean size and poly-dispersity Index (PDI) for three batches of exosomes and nanoparticles were reported. The same dispersion was transferred to a zeta potential cuvette for the measurement of the surface charge of the exosomes and nanoparticles.

### *In Vitro* Transfection Studies

A total of 2 × 10^6^ Panc-1 cells were seeded in T175 flasks in completed DMEM medium for transfection. Cells were transfected with plasmid DNA, which encapsulated by HA-PEI/HA-PEG in 1:9 ratio (%w/w) in DMEM with a dosage of 20 μg of plasmid DNA per 2 × 10^5^ cells and cultured at 37 °C. Naked plasmid DNA, Naked HA-PEI/HA-PEG nanoparticles, and plasmid DNA complexed with Lipofectamine^®^ 3000 were used as controls. After 6 h incubation, the media had been removed in all samples and replace fresh DMEM media. At 48 h post-transfection, the media was replaced by 10 μg/mL puromycin-containing DMEM for transfected cells stable selection. After 48 hours, the media were collected for exosomes isolation and purification, followed by exosomes RNA isolation. The cells were harvested for RNA isolation. Expression levels of miR-155 and miR-125b-2 genes in Panc-1 cells and exosomes were quantified by Taqman Gene Expression Assay with specific microRNA Taqman primer using quantitative PCR (qPCR) (LightCycler 480, Roche, Branford, CT). U6 was used as a housekeeping gene.

### PCR Analysis of Transfection and Exosome Content Modification

Real-time PCR was processed to identify the expression level of iNOS, IL-1β (endogenous M1 markers) and Arg1 (endogenous M2 marker) mRNA in J774A.1 macrophages for polarity determination in several macrophage reprogramming studies. The full details of each reprogramming study are presented in the Supplementary Materials. The amplifications were carried out on Applied Biosystems StepOnePlus™ System (Applied Biosystems). The comparative CT method was used to analyze the relative iNOS, IL-1β, and Arg1 mRNA expression levels. Expression of β-actin was used as an internal control.

### Data Analysis

Data were expressed as mean ± standard deviation. Statistical significance was determined by student’s t-test, with a one-tailed distribution. A probability (p) of less than 0.05 was considered statistically significant.

## Additional Information

**How to cite this article**: Su, M.-J. *et al*. Pancreatic Cancer Cell Exosome-Mediated Macrophage Reprogramming and the Role of MicroRNAs 155 and 125b2 Transfection using Nanoparticle Delivery Systems. *Sci. Rep.* **6**, 30110; doi: 10.1038/srep30110 (2016).

## Supplementary Material

Supplementary Information
