# Supplementary material for: Pancreatic Cancer Cell Exosome-Mediated Macrophage Reprogramming and the Role of MicroRNAs 155 and 125b2 Transfection using Nanoparticle Delivery Systems
Source: Sci Rep. 2016 Jul 22;6:30110. doi: 10.1038/srep30110 (PMC4957091; doi:10.1038/srep30110)
Supplement: Supplementary Information [file srep30110-s1.pdf]

Pancreatic Cancer Cell Exosome-Mediated Macrophage Reprogramming and the Role of MicroRNAs 155 and 125b2 Transfection using Nanoparticle Delivery Systems

Mei-Ju Su[1], Hibah Aldawsari[2], and Mansoor Amiji[1,2,3]

[1] Department of Pharmaceutical Sciences, School of Pharmacy, Northeastern University, Boston, MA 02115, USA
[2] Faculty of Pharmacy, King Abdulaziz University, Jeddah, Saudi Arabia

[3]*Corresponding author:*

Tel. (617) 373-3137
Fax (617) 373-8886
Email: m.amiji@neu.edu

Supplementary Information

Methods and Results

1. Evaluation of Baseline Macrophage Polarity and Establishment of Panc-1 and J774.A1 Cells in Co-culture System

1.1. Evaluation of Baseline Macrophage Polarity

To induce the pro-inflammatory subtype (M1), J774A.1 macrophages were cultured for 6 hours in the presence of 100 ng/mL IFN-γ and LPS. To induce the anti-inflammatory phenotype (M2), J774A.1 macrophages were culture for 6 hours in the presence of 100 ng/mL IL-4 for M2a phenotype, or 100 ng/mL IL-10/TGF-b for M2c phenotype.

1.2. RT-PCR Analysis of Macrophage Phenotype Modulation

1.2.1 Total RNA Extraction

The total RNA extraction was isolated from the cell lysates using the High Pure RNA Isolation kit. RNA extraction kit from all the cell pellets that were obtained from untreated J774A1, M1 polarized J774A1 for 6 hours, M1 polarized J774A1 for 72 hours, M1 polarized J774A1 cells co-culture with Panc-1for 72 hours, M2 polarized J774A1 for 6 hours conditions and quantified by using Nano Drop 2000. The extracted total RNA was stored at -80°C for further use.

1.2.2 RT-PCR Analysis of Macrophage Phenotype Expression Markers

The amplification of M1 macrophage marker, IL-1 beta gene and M2 macrophage markers, arginase-1 gene were performed on the extracted total RNA samples by using

the Verso® cDNA Synthesis Kit for first cDNA strand synthesis and Platinum TaqDNA® Polymerase for DNA amplification. Beta-actin was used as a housekeeping gene. The following are primer sequences, which were used for the amplification.

<u>β-actin</u>

Forward: 5'GCCTTCCTTCTTGGGTATGG 3'

Reverse: 5' CAGCTCAGTAACAGTCCGCC 3'

<u>INOS</u>

Forward: 5' CCTTGGTGAAGGGACTGAGC 3'

Reverse: 5' TGCTGTGCTACAGTTCCGAG 3'

<u>TNF- α</u>

Forward: 5' CATGAGCACAGAAAGCATGA 3'

Reverse: 5' CCTTCTCCAGCTGGAAGACT 3'

<u>IL-1 β</u>

Forward: 5' GGCTGCTTCCAAACCTTTGA 3'

Reverse: 5' GCTCATATGGGTCCGACAGC 3'

<u>IL-10</u>

Forward: 5' CCAAGCCTTATCGGAAATGA 3'

Reverse: 5' TCTCACCCAGGGAATTCAAA 3'

<u>Arginase</u>

Forward: 5' AGCACTGAGGAAAGCTGGTC 3'

Reverse: 5' TACGTCTCGCAAGCCAATGT 3'

Reverse transcription PCR was run on the thermo cycler (BioRad T100, Hercules, CA) for 2 min at 94 °C and 40 cycles of 94 °C for 30 sec, 55 °C for 30 sec, and 72 °C for 60 sec for all the primers. The amplified reaction products were visualized by running them on 2 % E Gels with SYBR Safe for 30 minutes. The gels were imaged by using ChemiDocTM XRS imaging system and the intensity or the density of the bands on the gels was quantified on the using ImageLab™ software on the system. Densitometric analysis of the bands was done to evaluate the expression levels of total RNA of the genes for specific M1/M2 macrophage endogenous markers.

1.3 Indirect Co-culture of Pancreatic Cancer Cells and Macrophage

Murine J774A.1 macrophages (2 x 10^5 cells/dish) were seeded in 100 mm polystyrene cell culture dishes in DMEM, supplemented with 10% FBS and 1% penicillin/streptomycin. Pancreatic cancer cells Panc-1 (3 x 10^5 cells/insert) were seeded into the upper chamber of a 75 mm Polycarbonate Transwell® Insert of 3.0 µm pore size in DMEM with 10% FBS and 1% penicillin/streptomycin. The following day, the J774A.1 macrophages were treated with IFN-γ and LPS at a concentration of 100 ng/ml for 6 hours, with the aim of promoting the classical or M1 phenotype of macrophages. After 6 hours, the media were replaced by new DMEM into the 100 mm cell culture dishes, and the culture inserts with Panc-1 cells were placed into the culture dishes containing J774A.1 macrophages, and incubated up to 72 hours in DMEM supplemented with 10% FBS and 1% penicillin/streptomycin. Cells were cultured in a standard humidified incubator at 37°C. After co-culture with Panc-1cell, the phenotype of J774A.1 cells had been analyzed by RT-PCR.

2. Dose-dependent Studies of J774A.1 Re-programming with Panc-1 Exosomes

The aim of the studies is to reprogram the J774A.1 macrophages via the effect of Panc-1 exosomes and MiR-155 modified Panc-1 exosomes. Murine J774A.1 macrophages (2 x 10^5 cells/dish) were seeded in 100 mm polystyrene cell culture dishes in DMEM. Cells were cultured in a standard humidified incubator at 37°C overnight. J774A.1 macrophages were cultured for a period of 6 hours in the presence of IFN-γ and LPS at a concentration of 100ng/ml, with the aim of promoting the classical or M1 phenotype of macrophages. After 6 hours, the media were replaced by new DMEM and different protein concentrations of exosomes, 40 μg, 80 μg, 120 μg, 160 μg, were added into the media for 72 hours. The J774A.1 macrophages were also treated with IL-4 and LPS/IFN-ϒ, respectively as positive control. The expression of M1 and M2 specific marker genes of J77A.1 macrophages at the mRNA level was then ascertained using two-step RT-PCR to determine phenotypic gene expression.

3. Macrophage Re-programming via MicroRNA-Mediated Re-programming Tumor Exosomes

Similar to dose-dependent studies of J774A.1 re-programming with Panc-1 derived exosomes, the aim of this study is to evaluate the effect of miR155/miR125b-2 modified exosomes on macrophage re-programming. Untreated J774A.1 macrophages were initially stimulated with 100ng/ml IL-4 to induce their polarization to M2 phenotype in each group. The miR155 and miR125b-2 modified exosomes were collected from plasmid DNA transfected panc-1 cells via HA-PEI/HA-PEG nanoparticles using same transfection protocol. The M2 states J774A.1 macrophages were further co-cultured with 160 μg of miR155/miR125b-2 modified exosomes in a standard humidified incubator at

37°C for 48 hours. After 48 hours, the J774A.1 macrophages were collected for evaluating the polarization states via specific M1/M2 endogenous markers. The expression of IL-1β, iNOS, and Arginase were then determined by qPCR using the ΔΔCt Method. The LightCycler® 480 SYBR Green I Master Mix was used for preparing various reactions and β -Actin was used as an endogenous control. The Roche Light Cycle 480 Instrument was used for running the reactions.

4. Macrophage Re-programming via MicroRNA-Mediated Re-programming Tumor Cells in Indirect Co-culture System

The aim of this study is to see if miR155/miR125b-2 modified Panc-1 cells co-culturing with J774A.1 macrophages has the same effect as miR155/miR125b-2 modified exosomes on macrophages re-programming. Using the same protocol for panc-1 cells and J774A.1 macrophages in transwell co-culture system, untreated J774A.1 macrophages were stimulated with 100ng/ml IFN-γ and LPS for 6 hours to induce their polarization to M1 phenotype. The transwell inserts containing 3 x 10^5/insert panc-1 cells were placed into the culture dishes containing J774A.1 macrophages, and incubated up to 48 hours to enable the macrophages polarize to M2 state. The miR155/miR125b-2 modified panc-1 cells were seeded on new transwell inserts by previous transfection protocol, and were used to replace the transwell insert containing non-modified panc-1 cells in co-culture system for 48 hours. The effect of miR155/miR125b modified panc-1 cells on the expression of M1 and M2 phenotype specific marker genes in J774A.1 macrophages polarization state was then assessed using qPCR followed by the same protocol in the previous study.

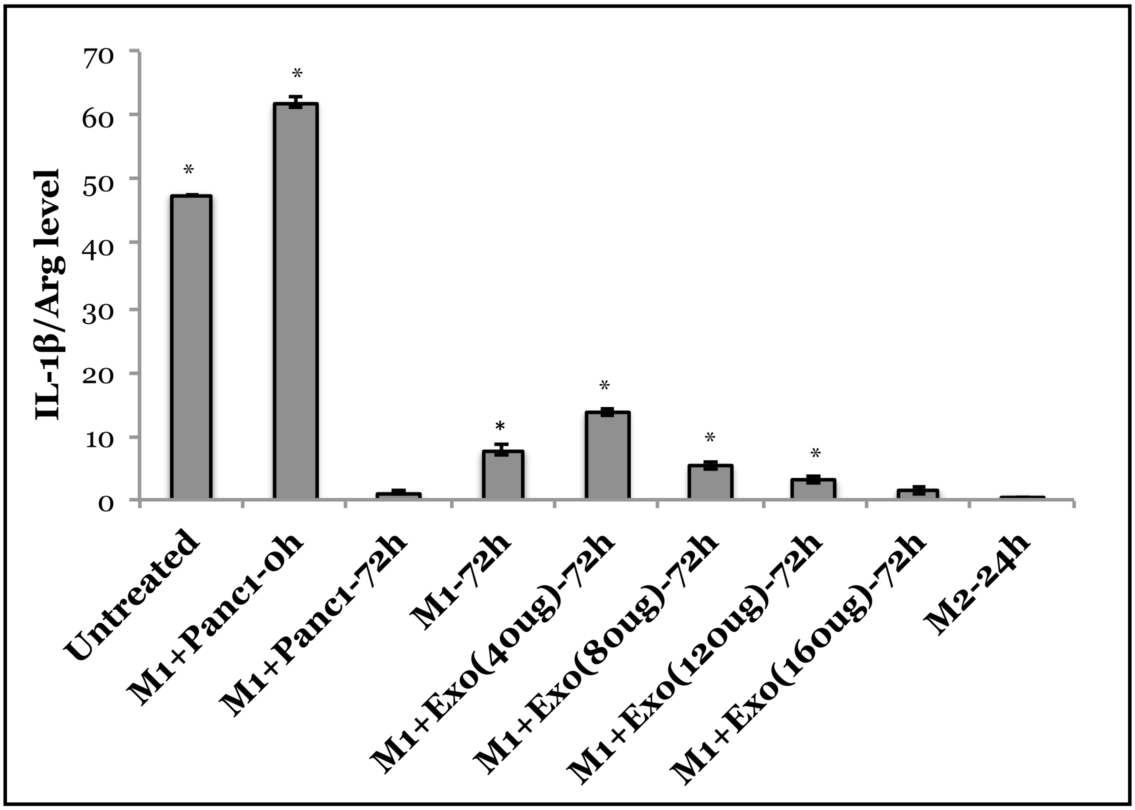

Supplementary Figure 1. Dose-dependent studies of J774A.1 re-programming with Panc-1 exosomes
IL-1β/Arg (M1/M2) level was used to evaluate the polarity of J774A.1 macrophages. The higher value of the ratio indicated the majority of macrophages were in M1 state. The J774A.1 macrophages, which didn't receive any treatment, were labeled "untreat" and the J774A. 1 macrophages, which received LPS and IFN- γ for 6h were labeled " M1+Panc-1-0h" in the figure. The macrophages were polarized to M1 state and futher co-cultured with panc-1 cells for 72h were labeled "M1+Panc-1-72h" in the figure. The macrophages were polarized to M1 state and further treated with 40, 80, 120, 160ug of panc-1 derived exosomes were labeled "M1+EXO-72h" with its exosomes protein concentration. The J774A.1 macrophages that received IL-4 for 24h were labeled "M2-24h" as the positive control in the figure.The IL-1β/Arg1 level decreased while the higher protein concentration of Panc-1exosomes was given to the macrophages. Dosing 160 ug of exosomes into J774A.1 macrophages in "M1+EXO 160ug-72h" group presented a similar value of the IL-1β/Arg1 ratio comparing to the "M1+Panc-1-72h" co-culture group. n = 3, *$p < 0.05$ compared to M1 macrophage co-culture with panc-1 cells group.

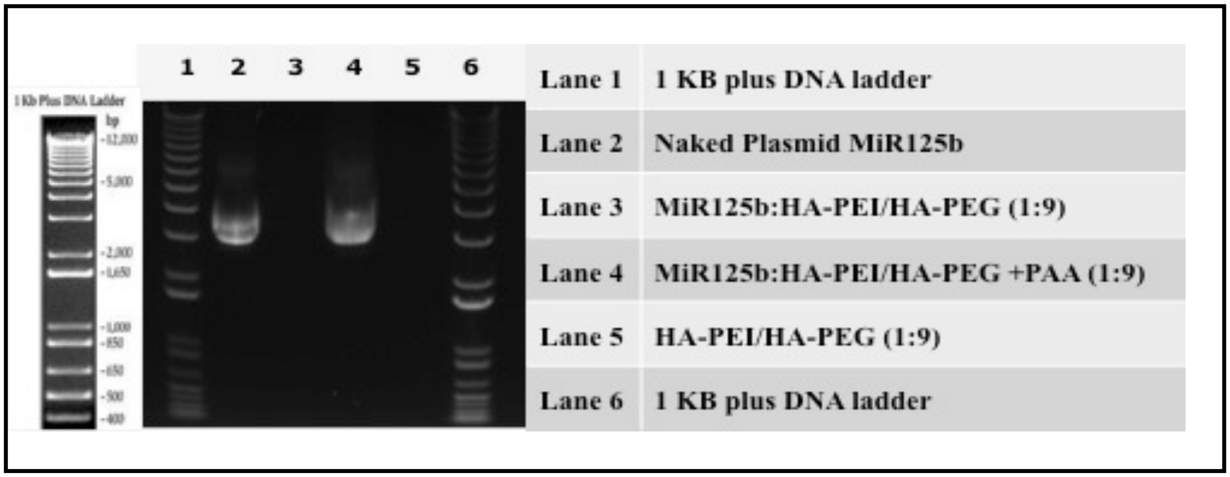

Supplementary Figure 2. Agarose gel electrophoresis analysis of plasmid encapsulation in HA-PEI/HA-PEG nanoparticles

The gel showed that naked plasmid miR-125b-2 wasn't degraded in lane 2. No band was presented in lane 3 and 2% PAA was used for de-complexation and release of the plasmid from the nanoparticles was shown in lane 4. Blank HA-PEI/HA-PEG NPs were load in lane 5.

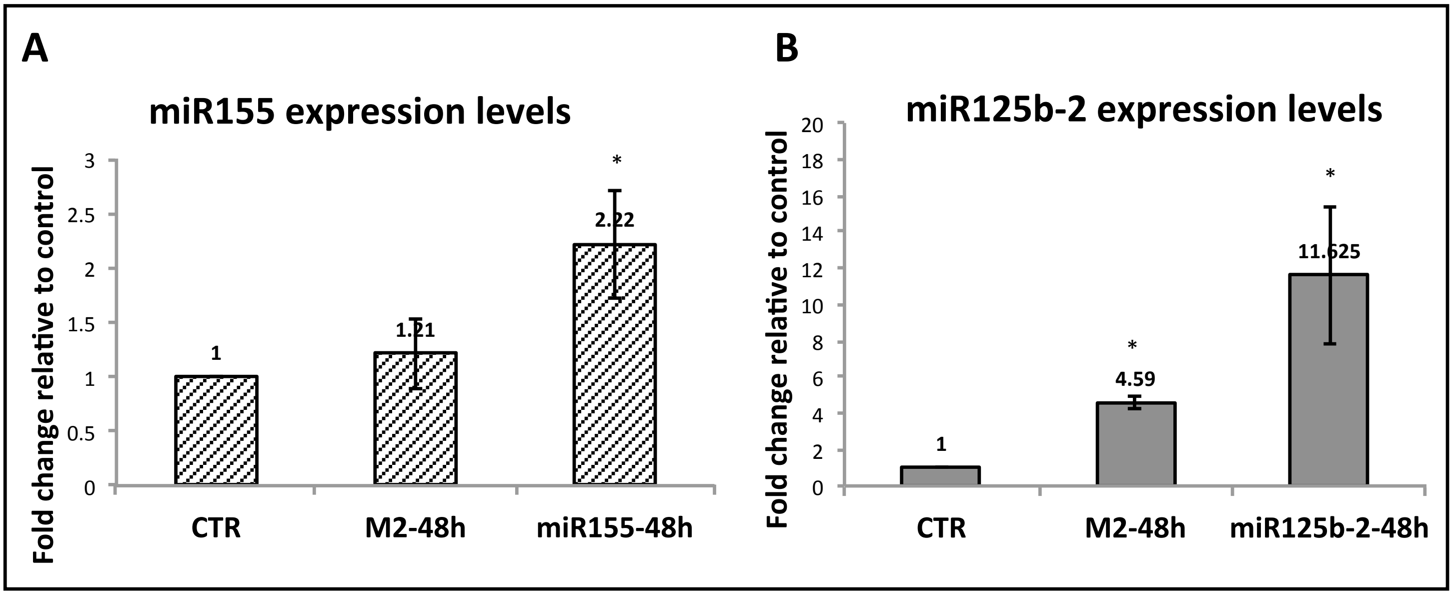

Supplementary Figure 3. Expression level of target gene in J774A.1 macrophages with 160 µg miR155/miR125b-2-modified exosomes. A. miR155 expression level in untreated and J774A.1 macrophages treated with 160 µg miR155 modified exosomes. B. miR125b-2 expression level in untreated and J774A.1 macrophages treated with 160 µg miR125b-2 modified exosome. . n = 3, *$p < 0.05$ compared to untreated macrophages.
